# Comparison of the Swedish STarT Back Screening Tool and the Short Form of the Örebro Musculoskeletal Pain Screening Questionnaire in patients with acute or subacute back and neck pain

**DOI:** 10.1186/s12891-017-1449-9

**Published:** 2017-02-21

**Authors:** Malin Forsbrand, Birgitta Grahn, Jonathan C. Hill, Ingemar F. Petersson, Charlotte Post Sennehed, Kjerstin Stigmar

**Affiliations:** 1 Lund University, Faculty of Medicine, Department of Clinical Sciences Lund, Orthopedics, Lund, Sweden; 2 Epidemiology and Register Centre South, Region Skåne, Lund, Sweden; 3 Blekinge Centre of Competence, Landstinget Blekinge, SE-371 81 Karlskrona, Sweden; 4 Department of Research and Development, Region Kronoberg, Växjö, Sweden; 5 Institute of Primary Care and Health Sciences, Keele University, Stoke-on-Trent, United Kingdom; 6 Department of Health Sciences, Physiotherapy, Lund University, Lund, Sweden

**Keywords:** Back pain, Neck pain, Validity, Primary care, Psychological factors

## Abstract

**Background:**

Patients with back and neck pain are often seen in primary care and it is important to provide them with tailored interventions based on risk stratification/triage. The STarT Back Screening Tool (SBT) is a widely used screening questionnaire which has not yet been validated for a population with back and/or neck pain with short duration. Our aim was to compare the concurrent validity of the SBT and the short form of the ÖMPSQ including psychometric properties and clinical utility in a primary care setting.

**Methods:**

Patients who applied for physiotherapy by direct access (January 2013 to January 2014) at 35 primary care centers in south Sweden, with acute or subacute back and/or neck pain, aged 18–67 years, who were not currently on sick leave or had been on sick leave less than 60 days were asked to complete the SBT and ÖMPSQ-short questionnaire (*n* = 329). We used the Spearman’s rank correlations to study correlations, cross tabulation and Cohen’s kappa to analyze agreement of patient classification. Clinical utility was described as clinician scoring miscalculations and misclassifications of total and/or subscale scores.

**Results:**

Completed SBT (9-items) and ÖMPSQ-short (10-items) data were available for 315/329 patients respectively. The statistical correlation for SBT and ÖMPSQ-short total scores was moderately strong (0.62, *p* < 0.01). In subgroup analyses, the correlations were 0.69 (*p* < 0.01) for males and 0.57 (*p* < 0.01) for females. The correlations were lower among older age groups, especially females over 50 years (0.21, *p* = 0.11). Classification to high or low risk for long-term pain and disability had moderate agreement (κ = 0.42). Observed classification agreement was 70.2%. The SBT had fewer miscalculations (13/315) than the ÖMPSQ-short (54/315).

**Conclusions:**

The correlation between the SBT and the ÖMPSQ-short scores were moderately strong for individuals with acute or subacute back and/or neck pain. SBT seemed to be clinically feasible to use in clinical practice. We therefore suggest that SBT can be used for individuals with both BP and/or NP in primary care settings but it is important to be aware of that SBT’s agreement with the ÖMPSQ-short was poor among females aged over 50 years.

**Trial registration:**

ClinicalTrials.gov ID: NCT02609750 Registered: November 18, 2015.

## Background

Musculoskeletal disorders are very common in the general population worldwide [1–3] causing disability for the individual and high costs for the society [4–6] The Global Burden of Disease study reported in 2012 that low back pain and neck pain (NP) was one of five top ranked causes for years lived with disability [7] and in European countries, individuals with back and NP constitute a large proportion of health care seeking in primary care [8, 9]. Low back pain increases the risk of general muscle pain, spinal pain and multiple health complaints [10] and to have low back pain together with neck-shoulder pain is associated with higher risk of long-term sickness absence [11].

About 85% of all low back pain and NP is classified as nonspecific, where the specific underlying disease or pathology remains unknown [12]. Most individuals with acute low back pain usually improve rapidly and return to work within 1 month [13] but after 12 months, about 60% still experience relapses of pain [14]. In a Swedish cohort of individuals seeking care for nonspecific low back pain or NP about half of the population reported pain and disability 5 years after onset [15].

Back pain (BP) is multi-factorial in both etiology and management [16]. Treatments should therefore be tailored based on the patient’s needs [17] and also equally distributed in relation to the patient’s needs [18]. A wide range of treatment options are available within primary care [19, 20] but there is still insufficient knowledge on how to direct individuals with BP to the right treatment option at the right time [21, 22] and how to prevent acute BP and NP from becoming chronic [23]. Consequently, a clinical and research priority is to, at an early stage, identify subgroups of patients with nonspecific BP and NP who are at risk of developing long-standing disability, in order to optimize treatment [17, 24].

Psychological risk factors have a key role in the transition from acute to chronic pain and the development of long-term disability [25–27]. The STarT Back Screening Tool (SBT) [28] is a validated risk stratification tool that includes questions on modifiable physical and psychosocial risk factors for long-term BP, in order to match individuals to appropriate treatments according to their prognostic profile. Patients are classified into three risk groups; low, medium or high risk for long-term pain and disability. Patients at low risk of poor outcome are directed to supported self-management, education and advice including pain relief, encouragement to stay active and are also informed about an overall good prognosis. Those at medium risk are offered evidence-based physiotherapy interventions such as manual therapy and exercise. For those at high risk, a combined physical and psychological approach is recommended [29]. Using the SBT together with targeted treatments has shown improved efficiency regarding patients’ clinical outcomes and reduced health care costs in the United Kingdom [30]. The SBT’s psychometric properties have been tested in several countries and it is now used in a number of different international settings [31–35]. The SBT was recently cross-culturally adapted and validated in Swedish in a small low back pain population (*n* = 62) [36].

The Örebro Musculoskeletal Pain Screening Questionnaire (ÖMPSQ) aims to identify patients at risk for developing work disability due to BP and NP [37]. ÖMPSQ is one of the most widely used screening questionnaires and several studies demonstrate the utility of the ÖMPSQ, both in research and clinical settings [38–41]. The ÖMPSQ was therefore considered the most appropriate reference standard against to validate the SBT. A short form of the ÖMPSQ (ÖMPSQ-short) has been developed to further increase the clinical utility of the ÖMPSQ [42]. The ÖMPSQ-short has earlier been compared with the SBT for patients with low back pain [33, 36, 43] but not yet for a population with patients applying for physiotherapy treatment due to BP and/or NP. The SBT has neither been compared or validated against the ÖMPSQ-short nor for a large primary care population in Sweden. Back and neck pain is common symptom in the general population and the pain from the back and neck often occurs concurrently [44]. Therefore clinicians need brief, practical tools for both BP and NP to identify patients at risk of poor outcome in order to decide the most appropriate interventions at an early stage [28, 42]. Since there is a lack of short and clinically useful instruments to guide clinicians in the management of patients with non-specific BP and/or NP, more research is needed. Therefore the overall aim of this study was to study the concurrent validity of the Swedish version of the SBT and the ÖMPSQ-short in a population of patients with acute or subacute BP and/or NP in a primary care setting. In addition, to investigate the agreement regarding classification into risk groups between the SBT and the ÖMPSQ-short and also to describe clinical utility of the two instruments.

## Methods

We conducted a cross-sectional study, nested within an ongoing clinical trial including patients with acute or subacute BP and/or NP (ClinicalTrials.gov ID: NCT02609750).

### Subjects

Patients were consecutively recruited between January 2013 and January 2014 from 35 primary care centers in the southern parts of Sweden. We asked patients that applied for physiotherapy due to an episode of acute or subacute nonspecific BP and/or NP, to participate in the study. Patients that were between 18 and 67 years, who were not currently on sick leave or had been on sick leave less than 60 days, completed SBT and ÖMPSQ-short at the first visit to the physiotherapist. Patients that were pregnant, had severe pathology (“red flags”) [19] or were not able to speak and understand Swedish, were not included. Red flags were screened for in a structured way [19] in connection to the clinical trial. If there were medical conditions in urgent need for medical care or examination, patients were referred to a doctor without delay. All patients applied for physiotherapy services through direct access. In all, 329 patients were identified.

### Procedure

All participants completed the SBT and ÖMPSQ-short at the same time at their first physiotherapy session. The questionnaires were scored by the physiotherapist according to the methods specified by the instrument developers [28, 37]. Data from the SBT and the ÖMPSQ-short questionnaires were manually entered into a SPSS 22.0 database and were thoroughly checked and validated. Patients were excluded if they had any missing item on SBT [28] and for the ÖMPSQ-short, missing items were handled as described by the original ÖMPSQ [45], where one missing item was accepted. The physiotherapist’s calculation of total score of SBT and ÖMPSQ-short were independently checked and errors corrected. All miscalculations were saved.

### Instruments

The SBT is a 9-item questionnaire including questions on known modifiable physical (item 1–4) and psychosocial (item 5–9) risk factors for long-term disabling BP, designed to support clinicians in directing individuals to different levels of care [28]. Item 1–4 is about referred leg pain, neck or shoulder pain, difficulties in walking and difficulties in dressing. Item 5–9 form the psychosocial subscale which screen for fear of physical activity, anxiety, pain catastrophizing, depressive mood and overall impact from their BP. The SBT total scores range between 0 and 9. Items 1–8 have a dichotomous response option; “disagree” (0p) or “agree” (1p). Item 9 uses a 5-point Likert Scale from “not at all” to “extremely”, where responses “very much” or “extremely” are counted as one point and the other responses as zero. A total SBT score of ≤3 points indicates low risk, and patients with a total score ≥4 points in combination with <4 points on the psychosocial subscale (item 5–9) are at medium risk. A psychosocial subscale score of ≥4 points indicates high risk for chronicity [28].

The ÖMPSQ-short is a 10-item questionnaire with questions about psychosocial risk factors for work disability due to the pain [42]. The ÖMPSQ-short is based on the original ÖMPSQ [37] and covers 2 items from each of five concept areas: pain (item 1–2), self-perceived function (item 3–4), distress (item 5–6), return to work expectancy (item 7–8) and fear avoidance beliefs (item 9–10). Item number 1 (duration of pain) has 10 categories, ranging from 0 to 1 week to more than 52 weeks, scoring is from 1 to 10 points. Item number 2–10 is rated from 0 to 10 point on a scale anchored by extremes, for example, “completely disagree” to “completely agree” or “no pain” to “pain as bad as it could be”. Item number 3, 4 and 8 has reversed scoring. A total score is calculated (range 1–100) where 1 to 50 points indicate low risk and 51 to 100 points indicate higher estimated risk for future work disability and higher levels of pain [42].

### Statistical analyses

SPSS 22.0 was used for all analyses. We used a non-parametric approach which was chosen based on the distribution of the data. We used the Spearman’s rank correlation coefficient to study the correlations between the SBT total scores with the ÖMPSQ-short total scores. We also conducted subgroup analyses, based on pain sites reported by the patients, gender and age. For pain sites, we divided the population in two groups based on the answer on question number two in SBT, which is about neck or shoulder pain. All patients who reported neck or shoulder pain were allocated to the NP + BP group (a mixed group of patients with neck or shoulder pain with or without BP). Patients who didn’t report neck or shoulder pain were allocated to the BP group and were regarded as having BP only. The reason for not analyzing patients with neck pain only was that we had no possibilities to identify them as we didn’t have access to the diagnoses of the patients. For gender, we divided the study population into females and males and for age, we divided the population in three age groups (≤39, 40–49 and ≥50 years). We found these age groups clinically relevant to study as the age 40–49 years is a period of life often associated with higher demands both at home and at work and might therefore result in a higher sick leave. A correlation coefficient less than 0.3 was considered as poor, 0.3–0.5 as fair, 0.6–0.8 moderately strong and greater than 0.8 was considered very strong [46].

We used cross-tabulation to describe the observed agreement, regarding classification into risk groups, between the ÖMPSQ-short (low and high risk) and the SBT (low, medium and high risk). To allow an agreement analysis of the SBT and the ÖMPSQ-short risk classifications, we did two analyses. First we merged the low and medium risk group for the SBT in the first analysis and then we merged the medium and high risk group for the SBT in a second analysis. We chose to present the results of the second analysis, in line with Fuhro et al. [43] as this appeared to be the most clinically relevant solution [28]. The agreement between classifications to either high or low risk by the two questionnaires was analyzed using Cohen’s kappa test where <0.20 were considered as poor agreement, 0.21 to 0.40 fair agreement, 0.41 to 0.60 moderate agreement, 0.61 to 0.80 good agreement, and values over 0.80 very good agreement [47]. The proportion of observed agreement was calculated by percentage. The McNemar test was used to determine if there were differences regarding allocation to the “low” or “high” risk group by the two instruments and to determine if the disagreement observed was balanced or skewed towards the lower or higher risk group.

We described the clinical utility of the two instruments as screening tools from a clinician’s perspective. Clinical utility was described as clinician scoring miscalculations and misclassifications of total and/or subscale scores of the two instruments. First, we calculated the number of physiotherapist scoring miscalculations of ÖMPSQ-short total scores and SBT total and subscale scores. Then, we calculated the miscalculations that had led to a misclassification. To analyze if a miscalculation of a total score had led to a misclassification to a higher or lower risk group, we used the cut-off scores specified by the instrument developers [42, 48] with three risk groups in the SBT (low, medium and high) and two risk groups of the ÖMPSQ-short (low and high) [42].

## Results

### Study population

**Fig. 1:**
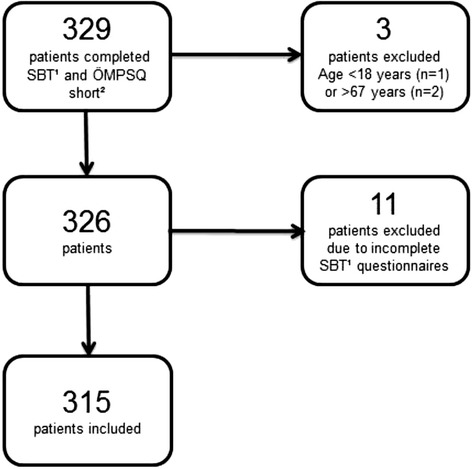
Flow chart of inclusion and exclusion of patients. ^1^Start Back Screening Tool. ^2^The Short Form of the Örebro Musculoskeletal Pain Screening Questionnaire

The flow chart of inclusion and exclusion of patients through the study and reasons for exclusion is presented in Fig. 1. There were 329 patients who consented to participate in the study and all patients completed the SBT and the ÖMPSQ-short questionnaires at the first physiotherapy session. Three patients didn’t meet the inclusion criteria of working age and were therefore not included in the study. Eleven patients were excluded because of missing items on the SBT questionnaires. In all, 315/329 patients (96%) were included in the final sample including 197 females (62.5%) and 118 males (37.5%).

Baseline characteristics of the study population are summarized in Table 1. In the BP population (*n* = 121) 51.2% were females and 48.8% were males. In the NP + BP population (*n* = 194) 69.6% were females and 30.4% were males. Among females (*n* = 197) 68.5% reported neck pain. Median age was 45 (range 20–66) for both females and males. The median scores (range) for SBT and ÖMPSQ-short were 4 (0–9) and 46 (9–81) respectively (Table 1).

**Table 1:** Characteristics of the study population, *n* = 315

	Number	Percent	Median	Range
Age				
Total population	315		45	20–66
≤39 years	108	34.3		
40–49 years	105	33.3		
≥50 years	102	32.4		
Gender				
Female	197	62.5		
Male	118	37.5		
Pain site^a^				
BP^b^	121	38.4		
NP + BP^c^	194	61.6		
*SBT* ^d^ total score, 0–9	315		4	0–9
Low risk	146	46.3		
Medium risk	133	42.2		
High risk	36	11.4		
*ÖMPSQ-short* ^e^ total score, 0–100	315		46	9–81
Low risk	200	63.5		
High risk	115	36.5		

^a^ *Pain site* Based on question number 2 (Neck or shoulder pain) on SBT, ^b^BP Back pain, ^c^NP + BP Patients with neck or shoulder pain (NP) with or without back pain (BP), ^d^ *SBT* Start Back Screening Tool, ^e^ *ÖMPSQ-short* Short form of the Örebro Musculoskeletal Pain Screening Questionnaire

### Correlations

**Fig. 2:**
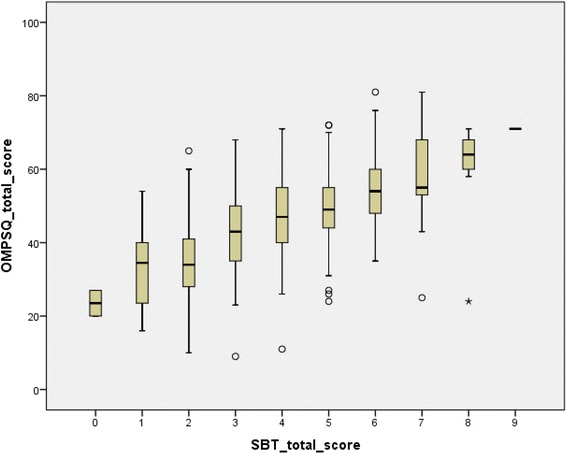
Box plot graph of the ÖMPSQ-short scores against the SBT total scores, *n* = 315, *r* = 0.62. *Asterisk* (*) represents extreme values. One person scored 9 points on SBT

The Spearman’s rank correlation coefficient for the SBT total scores and the ÖMPSQ-short scores was 0.62 (*p* < 0.01) which was considered as moderately strong (Fig. 2). The correlation between the SBT total scores and the ÖMPSQ-short scores for patients with BP (0.63, *p* < 0.01) and for patients with NP + BP (0.60, *p* < 0.01) was also moderately strong.

A summary of the statistical correlations for subgroup analyses, based on gender and age, is presented in Table 2. In subgroup analyses we found that the correlation for females was fair (0.57, *p* < 0.01) and for males it was moderately strong (0.69, *p* < 0.01). For participants aged ≤39 years, the correlation was moderately strong (0.72, *p* < 0.01) and for the group between 40–49 years (0.57, *p* < 0.01) and ≥50 years (0.50, *p* < 0.01), the correlation was fair. In further subgroup analyses, when we combined gender and age, we found the correlation for males ≥50 years moderately strong (0.71, *p* < 0.01) and for females ≥50 years poor (0.21, *p* = 0.11).

**Table 2:** Spearman correlation coefficient between SBT^a^ and ÖMPSQ-short^b^ total scores, *n* = 315

Population	Males and Females			Females			Males		
	*n*	*r*	*p*	*n*	*r*	*p*	*n*	*r*	*p*
Total population	315	0.62	<0.01	197	0.57	<0.01	118	0.69	<0.01
BP^c^	121	0.63	<0.01	62	0.58	<0.01	59	0.62	<0.01
NP + BP^d^	194	0.60	<0.01	135	0.56	<0.01	59	0.68	<0.01
≤39 years	108	0.72	<0.01	69	0.73	<0.01	39	0.75	<0.01
40–49 years	105	0.57	<0.01	71	0.60	<0.01	34	0.50	<0.01
≥50 years	102	0.50	<0.01	57	0.21	0.11	45	0.72	<0.01

^a^ *SBT* Start Back Screening Tool, ^b^ *ÖMPSQ-short* Short form of the Örebro Musculoskeletal Pain Screening Questionnaire, ^c^ *BP* Back Pain, ^d^ *NP + BP* Patients with neck or shoulder pain (NP) with or without back pain (BP)

### Observed agreement

The purpose of the SBT and ÖMPSQ-short was to identify patients that are at risk for long-term disability and in need for extended treatment. The observed agreement between the two questionnaires subgroup classification is shown in Table 3. The SBT classified 53.7% as high risk and 46.3% as low risk while the ÖMPSQ-short classified 36.5% as high risk and 63.5% as low risk (Table 3). The proportion of total observed agreement between the two questionnaires regarding participant classification to low and high risk for long-term pain and disability was 70.2%. Thus, 29.8% was allocated in disagreement. Participant classification by both questionnaires had moderate agreement (κ = 0.42, *p* < 0.01). We found differences regarding classification into low and high risk subgroups (McNemar, *p* < 0.01). The disagreement observed (29.8%) was significantly skewed towards the high risk group with a higher proportion of patients allocated to the SBT high risk group (53.7%) than to the ÖMPSQ-short high risk group (36.5%).

**Table 3:** Observed agreement of SBT^a^ (high risk = medium + high risk) and ÖMPSQ-short^b^ subgroups, *n* = 315

ÖMPSQ-short	SBT		
	Low risk	High risk	Score
Low risk	126	74	200
High risk	20	95	115
Score	146	169	315

^a^ *SBT* Start Back Screening Tool, ^b^ *ÖMPSQ-short* Short form of the Örebro Musculoskeletal Pain screening questionnaire

### Clinical utility

We studied the clinical utility from a clinician’s perspective. Physiotherapists had done more miscalculations on total scores in the ÖMPSQ-short (54/315) than in the SBT (13/315). In the SBT questionnaires we found 22 miscalculations of the SBT subscale scores. Among the miscalculations of total scores, seven of the ÖMPSQ-short questionnaires and five of the SBT questionnaires, led to misclassifications to a higher or lower risk group. In four respectively 21 SBT questionnaires, total scores and subscale score calculations were missing. First author (MF) calculated these scores. There were no missing calculations of total scores in the ÖMPSQ-short.

## Discussion

This is the first time that the SBT has been compared with the ÖMPSQ-short for a large group of patients with acute or subacute BP and/or NP in primary care in Sweden. This study demonstrated moderately strong correlations between the SBT and ÖMPSQ-short total scores for individuals with back and/or neck pain with short duration. The observed classification agreement was 70.2%. In subgroup analyses, we found the correlation was lower among females than for males and also lower among older age groups, especially females aged over 50 years. Clinicians made less miscalculations with the SBT compared to the ÖMPSQ-short.

The moderately strong correlation between SBT and ÖMPSQ-short total scores (0.62) in this study is similar to the results in a previous Swedish study (0.61) [36] and a Brazilian study (0.73) [43] that also compared SBT and ÖMPSQ-short total scores. Both these studies were in a low back pain population. Higher correlations have been reported when comparing the SBT with the original ÖMPSQ (24 item) for patients with low back pain in an English population (0.80) [49] and in a French population (0.74) [31] but also lower correlations have been found in a Finnish population (0.45) [33]. Cross-cultural differences may be one factor that can explain the differences between different study results. In contrast to the above mentioned studies, the study population in this study also includes patients that can have NP, but this does not seem to affect the correlations substantially.

In the subgroup analyses, we found the correlation was lower in females than for males and was further reduced among increasing age groups. We have also used other age groups in the analyses but regardless of which type of age group we used, we found the same results. We unexpectedly found the correlation for females ≥50 years as poor while the correlation for males ≥50 years were still moderately strong. We can’t rule out that this difference between males and females ≥50 years might be random but there may also be biological differences. There is a gender gap concerning low back pain, where females are having more prevalent comorbidity of neck and shoulder pain and psychosocial distress than males [44]. In this study there were more females than males and a greater share of females that had neck pain (68.5 vs 50%). On the other hand, when studying different age groups, a greater share of younger males had neck pain (51.3%) compared to the oldest age group (44.4%). This difference was not seen in females. In other studies, comparing the SBT and the ÖMPSQ, the percentage of females varies between 55.6 and 80.8% but no results on the percentage of participants with neck pain, nor subgroup analyses for correlation based on gender and age have been reported in these studies [31, 33, 36, 43, 49].

Participant classification to low or high risk for long-term pain and disability by the SBT and the ÖMPSQ-short questionnaires had moderate agreement (κ = 0.42). Similar results were also found in the Brazilian study (κ = 0.49) [43] and in a study where they compared the SBT with the original ÖMPSQ (24 item) in an English population (κ = 0.57) [49]. The English study also showed significant differences between the SBT and the ÖMPSQ (24-item) regarding the threshold for high risk. The proportion of patients allocated to the high risk group in our study was higher for the SBT (53.7%) than for the ÖMPSQ-short (36.5%) which also was found by Fuhro et al. [43]. In contrast, Hill et al. [49] found that the SBT allocated fewer (35%) patients to the high risk group than the original ÖMPSQ (24-item) (38%). The 24-item ÖMPSQ has three risk groups, as the SBT. The higher proportion of patients allocated to the high risk group by the SBT in our study might have been influenced of the high risk classification we used when we merged the medium and high risk group for the SBT, but this method was used both in our and in the Brazilian study [43] but not in the UK-study [49]. Thus, when clinicians use the SBT instead of the ÖMPSQ-short, they will likely find more patients identified as medium or high risk by the SBT compared to the ÖMPSQ-short.

When choosing a classification instrument, clinicians and organizations need to be aware of, that patients at medium and especially patients at high risk need a more extensive treatment compared to those at low risk who can be reassured and offered less intensive treatment [29]. Patients at high risk are especially important to identify at an early stage as this group of patients will benefit most from psychological informed physiotherapy approaches. But, it is also important to be aware of the potential of misclassifying high risk and that there may be patients not being high risk and not being appropriate for the enhanced intervention approach.

However, patients with medium and high risk are those who will benefit most from physiotherapy [30] and to identify them at an early stage, will maximize treatment benefit, reduce harm and increase health-care efficiency by offering the right treatment to the right patient at the right time [17].

To the best of our knowledge, this is the first time that clinical utility has been focused when comparing the two instruments. It was a difference regarding completion rates from patients with SBT having some incomplete questionnaires (*n* = 11) while the ÖMPSQ-short had none. One contributing factor might be the order in which the questionnaires were completed. Patients completed the ÖMPSQ-short first, as the ÖMPSQ-short was used as an inclusion criteria to the clinical trial (ClinicalTrials.gov ID: NCT02609750). The physiotherapists might have checked all questions and calculated the ÖMPSQ-short scores more carefully. Physiotherapists in Sweden are also more used to score the ÖMPSQ-short than the SBT. The rates of missing calculations of total and subscale scores in the SBT might be due to the instructions to the physiotherapists. They were not explicitly told to do these calculations. The fact that it was the treating physiotherapist that administered and scored the SBT and that they had different experiences of using the two questionnaires may have influenced the rates of miscalculations of scores. But, at the same time, this might have had minimal impact of the results as there were so many primary care centers (35) in different regions included. The higher rate of miscalculations of total scores in ÖMPSQ-short (54/315) compared to SBT (13/315) indicates that the SBT is easier to score for clinicians. A potential benefit of using the SBT instead of the ÖMPSQ-short might be that the SBT seems to be more clinical feasible to use in routine clinical practice**.** More miscalculations in the ÖMPSQ-short might be due to the more complex scoring with 0–10 points for each item and also the reversed scoring in three items (3, 4 and 8). However, even though there were more miscalculations of total scores in the ÖMPSQ-short than in the SBT, there were no difference in misclassification to either a higher or lower risk group between the ÖMPSQ-short (*n* = 7) and the SBT (*n* = 5) questionnaires. Consequently, miscalculations of the SBT and the ÖMPSQ-short total scores do not seem to substantially influence the risk classification. Clearly introducing electronic questionnaires with automatic summations have the potential to eliminate errors even further.

### Strengths and limitations

The main strength of the present study is the size of the study population (*n* = 315) at a great number (35) of different real world primary care settings. The same individuals completed both the SBT and ÖMPSQ-short at the same time. We have thoroughly checked and validated all questionnaires and also studied psychometric properties and clinical utility in a primary care setting. The population studied was relatively homogenous including only patients with short duration of pain, not participants with chronic pain. This means that we can identify patients at risk of poor prognosis at an early stage where it is still possible to do brief interventions and influence outcome by treating modifiable prognostic factors and stratify care.

To our knowledge, this is the first time SBT is validated in a population with both NP and BP. A limitation in this study is that we have no diagnoses registered for the study population and therefore we were not able to distinguish patients diagnosed with only NP. On the other hand, having NP with or without comorbid BP is common [44, 50] and thus makes the results of this study applicable to a common clinical situation. Another limitation was that we merged the medium and high risk group for the SBT in the analysis to allow a comparison with the ÖMPSQ-short. But regardless of merging the medium and high risk group or merging the low and medium risk group for the SBT, we found significant differences in observed agreement between the two instruments. The observed classification agreement was 70.2% but there was at the same time a disagreement rate of 29.8%. The agreement analysis strategy used in this study by merging the medium and high risk group for the SBT may limit the ability to evaluate for false positive or negative SBT medium risk classification. However, the findings in this study with a moderately strong correlation between the SBT and the ÖMPSQ-short scores support that the SBT can be used as a clinical tool for patients with acute and subacute BP and/or NP in primary care and that the SBT will provide clinicians with more additional guidance in the level of care compared to the ÖMPSQ-short. The SBT is designed for stratified care, which involves targeting treatment to subgroups of patients based on their key characteristics [17] and we think this is an advantage in primary care because clinical intuition does not always match consistently to patient prognosis [51]. When using stratified care, clinicians can minimize the risk of overtreatment for low risk patients and give more appropriate treatment for medium and high risk patients [30]. The SBT may help the physiotherapists, how to prioritize between different treatments pathways. Future studies are needed to study how the SBT can predict chronic pain and work disability for the target group of patients with BP and/or NP in primary care and especially in the group of elderly females.

## Conclusion

This study showed that the correlation between the SBT and the ÖMPSQ-short scores were moderately strong for individuals with acute or subacute BP and/or NP. SBT seemed to be clinically feasible to use in clinical practice.

We therefore suggest that SBT can be used for individuals with both BP and/or NP in primary care settings but it is important to be aware of that SBT’s agreement with the ÖMPSQ-short was poor among females aged over 50 years.

## Abbreviations

BP: Back pain
NP: Neck pain
ÖMPSQ: Örebro Musculoskeletal Pain Screening Questionnaire (24-item)
ÖMPSQ-short: A Short form of the Örebro Musculoskeletal Pain Screening Questionnaire (10-item)
SBT: STarT Back Screening Tool

## References

[1] Balague F, Mannion AF, Pellise F, Cedraschi C (2012). Non-specific low back pain. Lancet.

[2] Fejer R, Kyvik KO, Hartvigsen J (2006). The prevalence of neck pain in the world population: a systematic critical review of the literature. Eur Spine J.

[3] Hoy D, Bain C, Williams G, March L, Brooks P, Blyth F, Woolf A, Vos T, Buchbinder R (2012). A systematic review of the global prevalence of low back pain. Arthritis Rheum.

[4] Gustavsson A, Bjorkman J, Ljungcrantz C, Rhodin A, Rivano-Fischer M, Sjolund KF, Mannheimer C (2012). Socio-economic burden of patients with a diagnosis related to chronic pain--register data of 840,000 Swedish patients. Eur J Pain.

[5] Alexanderson K, Norlund A (2004). Aim, background, key concepts, regulations, and current statistics. Scand J Public Health Suppl.

[6] Hansson T, Jensen I (2004). Swedish Council on Technology Assessment in Health Care (SBU). Chapter 6. Sickness absence due to back and neck disorders. Scand J Public Health Suppl.

[7] Vos T, Flaxman AD, Naghavi M, Lozano R, Michaud C, Ezzati M, Shibuya K, Salomon JA, Abdalla S, Aboyans V (2012). Years lived with disability (YLDs) for 1160 sequelae of 289 diseases and injuries 1990–2010: a systematic analysis for the Global Burden of Disease Study 2010. Lancet.

[8] Kinge JM, Knudsen AK, Skirbekk V, Vollset SE (2015). Musculoskeletal disorders in Norway: prevalence of chronicity and use of primary and specialist health care services. BMC Musculoskelet Disord.

[9] Jordan KP, Kadam UT, Hayward R, Porcheret M, Young C, Croft P (2010). Annual consultation prevalence of regional musculoskeletal problems in primary care: an observational study. BMC Musculoskelet Disord.

[10] Hagen EM, Svensen E, Eriksen HR, Ihlebaek CM, Ursin H (2006). Comorbid subjective health complaints in low back pain. Spine.

[11] Nyman T, Grooten WJ, Wiktorin C, Liwing J, Norrman L (2007). Sickness absence and concurrent low back and neck-shoulder pain: results from the MUSIC-Norrtalje study. Eur Spine J.

[12] Deyo RA, Weinstein JN (2001). Low back pain. N Engl J Med.

[13] Pengel LH, Herbert RD, Maher CG, Refshauge KM (2003). Acute low back pain: systematic review of its prognosis. BMJ.

[14] Hestbaek L, Leboeuf-Yde C, Manniche C (2003). Low back pain: what is the long-term course? A review of studies of general patient populations. Eur Spine J.

[15] Enthoven P, Skargren E, Oberg B (2004). Clinical course in patients seeking primary care for back or neck pain: a prospective 5-year follow-up of outcome and health care consumption with subgroup analysis. Spine (Phila Pa 1976).

[16] Richmond J (2012). Multi-factorial causative model for back pain management; relating causative factors and mechanisms to injury presentations and designing time- and cost effective treatment thereof. Med Hypotheses.

[17] Foster NE, Hill JC, O’Sullivan P, Hancock M (2013). Stratified models of care. Best Pract Res Clin Rheumatol.

[18] The National Board of Health and Welfare S. God vård, (National indicators of good care) (in Swedish). https://www.socialstyrelsen.se/publikationer2009/nationellaindikatorerforgodvard. Accessed 6 Feb 2017.

[19] van Tulder M, Becker A, Bekkering T, Breen A, del Real MT, Hutchinson A, Koes B, Laerum E, Malmivaara A (2006). Chapter 3. European guidelines for the management of acute nonspecific low back pain in primary care. Eur Spine J.

[20] Koes BW, van Tulder M, Lin C-WC, Macedo LG, McAuley J, Maher C (2010). An updated overview of clinical guidelines for the management of non-specific low back pain in primary care. Eur Spine J.

[21] Foster NE, Hill JC, Hay EM (2011). Subgrouping patients with low back pain in primary care: are we getting any better at it?. Man Ther.

[22] Koes B (2011). Management of low back pain in primary care: a new approach. Lancet.

[23] SBU (2016). Acute neck and back pain: preventive interventions - Effects of physical training, manual treatment and cognitive behavioural interventions.

[24] Abbott A (2016). Evidence base and future research directions in the management of low back pain. World J Orthop.

[25] Pincus T, Burton AK, Vogel S, Field AP (2002). A systematic review of psychological factors as predictors of chronicity/disability in prospective cohorts of low back pain. Spine (Phila Pa 1976).

[26] George SZ, Beneciuk JM (2015). Psychological predictors of recovery from low back pain: a prospective study. BMC Musculoskelet Disord.

[27] Grotle M, Foster NE, Dunn KM, Croft P (2010). Are prognostic indicators for poor outcome different for acute and chronic low back pain consulters in primary care?. Pain.

[28] Hill JC, Dunn KM, Lewis M, Mullis R, Main CJ, Foster NE, Hay EM (2008). A primary care back pain screening tool: identifying patient subgroups for initial treatment. Arthritis Rheum.

[29] Sowden G, Hill JC, Konstantinou K, Khanna M, Main CJ, Salmon P, Somerville S, Wathall S, Foster NE (2012). Targeted treatment in primary care for low back pain: the treatment system and clinical training programmes used in the IMPaCT Back study (ISRCTN 55174281). Fam Pract.

[30] Hill JC, Whitehurst DGT, Lewis M, Bryan S, Dunn KM, Foster NE, Konstantinou K, Main CJ, Mason E, Somerville S (2011). Comparison of stratified primary care management for low back pain with current best practice (STarT Back): a randomised controlled trial. Lancet.

[31] Bruyere O, Demoulin M, Beaudart C, Hill JC, Maquet D, Genevay S, Mahieu G, Reginster JY, Crielaard JM, Demoulin C (2014). Validity and reliability of the French version of the STarT Back screening tool for patients with low back pain. Spine (Phila Pa 1976).

[32] Morso L, Albert H, Kent P, Manniche C, Hill J (2011). Translation and discriminative validation of the STarT Back Screening Tool into Danish. Eur Spine J.

[33] Piironen S, Paananen M, Haapea M, Hupli M, Zitting P, Ryynanen K, Takala EP, Korniloff K, Hill JC, Hakkinen A (2016). Transcultural adaption and psychometric properties of the STarT Back Screening Tool among Finnish low back pain patients. Eur Spine.

[34] Luan S, Min Y, Li G, Lin C, Li X, Wu S, Ma C, Hill JC (2014). Cross-cultural adaptation, reliability, and validity of the Chinese version of the STarT Back Screening Tool in patients with low back pain. Spine (Phila Pa 1976).

[35] Karstens S, Krug K, Hill JC, Stock C, Steinhaeuser J, Szecsenyi J, Joos S (2015). Validation of the German version of the STarT-Back Tool (STarT-G): a cohort study with patients from primary care practices. BMC Musculoskelet Disord.

[36] Betten C, Sandell C, Hill JC, Gutke A (2015). Cross-cultural adaptation and validation of the Swedish STarT Back Screening Tool. Eur J Physiother.

[37] Linton SJ, Hallden K (1998). Can we screen for problematic back pain? A screening questionnaire for predicting outcome in acute and subacute back pain. Clin J Pain.

[38] Westman A, Linton SJ, Ohrvik J, Wahlen P, Leppert J (2008). Do psychosocial factors predict disability and health at a 3-year follow-up for patients with non-acute musculoskeletal pain? A validation of the Orebro Musculoskeletal Pain Screening Questionnaire. Eur J Pain.

[39] Linton SJ, Boersma K (2003). Early identification of patients at risk of developing a persistent back problem: the predictive validity of the Orebro Musculoskeletal Pain Questionnaire. Clin J Pain.

[40] Hockings RL, McAuley JH, Maher CG (2008). A systematic review of the predictive ability of the Orebro Musculoskeletal Pain Questionnaire. Spine (Phila Pa 1976).

[41] Dagfinrud H, Storheim K, Magnussen LH, Odegaard T, Hoftaniska I, Larsen LG, Ringstad PO, Hatlebrekke F, Grotle M (2013). The predictive validity of the Orebro Musculoskeletal Pain Questionnaire and the clinicians’ prognostic assessment following manual therapy treatment of patients with LBP and neck pain. Man Ther.

[42] Linton SJ, Nicholas M, MacDonald S (2011). Development of a short form of the Orebro Musculoskeletal Pain Screening Questionnaire. Spine (Phila Pa 1976).

[43] Fuhro FF, Fagundes FR, Manzoni AC, Costa LO, Cabral CM (2016). Orebro Musculoskeletal Pain Screening Questionnaire Short-Form and STarT Back Screening Tool: correlation and agreement analysis. Spine.

[44] Leijon O, Wahlstrom J, Mulder M (2009). Prevalence of self-reported neck-shoulder-arm pain and concurrent low back pain or psychological distress: time-trends in a general population, 1990–2006. Spine (Phila Pa 1976).

[45] Linton SJ. Örebromanualen för screening av patienter med muskuloskeletala besvär : tidig identifiering av patienter i riskzonen för kronisk smärta. Örebro: Yrkes- och miljömedicinska kliniken, Regionsjukhuset i Örebro; 1999

[46] Chan YH (2003). Biostatistics 104: correlational analysis. Singapore Med J.

[47] Altman DG (1991). Practical statistics for medical research.

[48] Hay EM, Dunn KM, Hill JC, Lewis M, Mason EE, Konstantinou K, Sowden G, Somerville S, Vohora K, Whitehurst D (2008). A randomised clinical trial of subgrouping and targeted treatment for low back pain compared with best current care. The STarT Back Trial Study Protocol. BMC Musculoskelet Disord.

[49] Hill JC, Dunn KM, Main CJ, Hay EM (2010). Subgrouping low back pain: a comparison of the STarT Back Tool with the Orebro Musculoskeletal Pain Screening Questionnaire. Eur J Pain.

[50] Palacios-Cena D, Alonso-Blanco C, Hernandez-Barrera V, Carrasco-Garrido P, Jimenez-Garcia R, Fernandez-de-las-Penas C (2015). Prevalence of neck and low back pain in community-dwelling adults in Spain: an updated population-based national study (2009/10-2011/12). Eur Spine J.

[51] Hill JC, Vohora K, Dunn KM, Main CJ, Hay EM (2010). Comparing the STarT back screening tool’s subgroup allocation of individual patients with that of independent clinical experts. Clin J Pain.

